# The Efficacy and Safety of Intrasphincteric Botulinum Toxin-A Injections in Patients with Non-Spinal Cord Injury-Related Detrusor Sphincter Dyssynergia: A Retrospective Study

**DOI:** 10.3390/biomedicines11113016

**Published:** 2023-11-10

**Authors:** Kilian Röthlin, Ralf Anding, Helge Seifert, Margret Hund-Georgiadis, Sandra Möhr, Matthias Walter

**Affiliations:** 1 Department of Urology, University Hospital Basel, University of Basel, 4031 Basel, Switzerland; k.roethlin@hotmail.com (K.R.); ralf.andi@googlemail.com (R.A.); helge.seifert@usb.ch (H.S.); 2 Neuro-Urology, REHAB Basel, 4055 Basel, Switzerland; 3 Alta uro AG, Medical Center for Urology, 4051 Basel, Switzerland;; 4 Clinic of Neurorehabilitation and Paraplegiology, REHAB Basel, 4055 Basel, Switzerland; m.hund@rehab.ch

**Keywords:** botulinum toxin-A, detrusor sphincter dyssynergia, electromyography, external urethral sphincter, non-spinal cord injury

## Abstract

Botulinum toxin-A (BoNT-A) injections into the external urethral sphincter are an established therapeutic procedure for reducing bladder outlet obstruction in patients with detrusor sphincter dyssynergia (DSD) due to spinal cord injury (SCI). Given the paucity of data on patients with DSD but without SCI, we aimed to assess the efficacy of intrasphincteric BoNT-A injections in this cohort. For this retrospective study, we screened all patients who underwent their first intrasphincteric BoNT-A injection at our institution between 2015 and 2021. The inclusion criteria were patients aged 18 years or older with neurogenic detrusor overactivity (NDO) and DSD with a maximum detrusor pressure (Pdetmax) of >40 cmH_2_O, confirmed via video-urodynamic studies (VUDS). The primary outcome was a reduction in Pdetmax and detrusor overactivity leak point pressure (DOLPP) during NDO-associated urinary incontinence posttreatment. The secondary outcome was a reduction in patients relying on indwelling urinary catheters posttreatment. We included 13 eligible patients (all male, median age 31 years, with different underlying neurological disorders, except SCI). All underwent intrasphincteric BoNT-A injections with either 100 (n = 7) or 150 (n = 6) units, respectively. Pdetmax during voiding was significantly reduced posttreatment (median 105 vs. 54 cmH_2_O, *p* = 0.006), whereas DOLPP remained unchanged (i.e., median 50 cmH_2_O). While seven patients relied on indwelling urinary catheters pre-treatment, all were catheter-free posttreatment. Intrasphincteric BoNT-A injections in patients with non-SCI related DSD appear feasible for reducing bladder outlet obstruction to a certain degree in this cohort and subsequently for reducing the rate of indwelling catheters.

## 1. Introduction

Volitional urinary bladder emptying is a synchronized act between the contraction of the detrusor muscle and relaxation of the external urethral sphincter (EUS) muscle. Patients with a suprasacral spinal cord injury (SCI) are at risk for detrusor sphincter dyssynergia (DSD) [1]. In these patients, the coordination between the detrusor muscle and the EUS is not synchronized anymore, hence, both muscles contract at the same time during voiding [1]. This may lead to high intravesical pressures as a consequence of functional bladder outlet obstruction, which is also a risk factor for recurrent urinary tract infections (UTI), deterioration of the upper tract function, or autonomic dysregulation [1].

To date, external sphincterotomy is the standard therapeutic procedure in patients with DSD due to SCI for attenuating bladder outlet obstruction [2]. However, sphincterotomy has been reported to be associated with a frequent need to repeat surgeries (6–50%) or treatment failure (25–50%), as well as intra-/ postoperative bleeding requiring blood transfusion (5–23%) [3,4]. The injection of botulinum toxin-A (BoNT-A) into the EUS to paralyze the muscle (e.g., for achieving a temporary chemical sphincterotomy) was first described by Dykstra et al. in 1988 [5]. There are various studies that have demonstrated the feasibility [5,6,7,8,9,10,11,12,13,14,15] and therapeutic benefit [9,10,11,12,13,14,15] of this procedure in patients with DSD due to SCI and multiple sclerosis (MS) [16]. However, to our knowledge, there is a lack of evidence regarding the therapeutic effect of intrasphincteric BoNT-A injections in non-SCI patients with DSD. Therefore, our aim was to analyze the efficacy and safety of intrasphincteric BoNT-A injections in patients with non-SCI-related DSD.

## 2. Materials and Methods

### 2.1. Population

Patients were eligible when meeting the following inclusion criteria: an age of ≥18 years; the presence of non-SCI related DSD; the presence of neurogenic detrusor overactivity (NDO) with a maximum detrusor pressure (Pdetmax) of >40 cmH_2_O during video-urodynamic studies (VUDS); underwent first intrasphincteric BoNT-A injections between 2015 and 2021; and underwent a follow-up (video-)urodynamic study (V)UDS. Further, patients must not have had any spinal fractures or SCI, which were established using computed tomography or magnetic resonance imaging.

### 2.2. Objectives

The primary outcome was a reduction in Pdetmax and detrusor overactivity leak point pressure (DOLPP) during NDO-associated urinary incontinence posttreatment. The secondary outcome was a reduction in the number of patients relying on indwelling urinary catheters posttreatment.

### 2.3. Outcome Variables

We chose the following categorical outcome variables: sex (female and male), type of underlying neurological disorder/injury (brain injury: traumatic (TBI) or hypoxic; stroke, and cerebral palsy, etc.), dosage of BoNT-A (100 or 150 units), the presence of NDO (yes or no), the presence of Pdetmax during storage > 40 cmH_2_O (yes or no), the presence of DSD (yes or no), the presence of post-void residual (PVR) urine, the method of bladder emptying (indwelling catheter (transurethral/suprapubic), condom catheter, intermittent catheterization (i.e., self or assisted), or volitional voiding), and vigilance state (Glasgow Coma Scale (GCS) = 15, unresponsive wakefulness syndrome (UWS) or minimally conscious state (MCS)).

We chose the following continuous outcome variables: age (years), urodynamic parameters: i.e., maximum cystometric capacity (MCC, (mL)), PdetQmax (cmH_2_O), Pdetmax during voiding (cmH_2_O), and DOLPP, (cmH_2_O), PVR urine (mL).

### 2.4. Data Source and Collection

**Figure 1:**
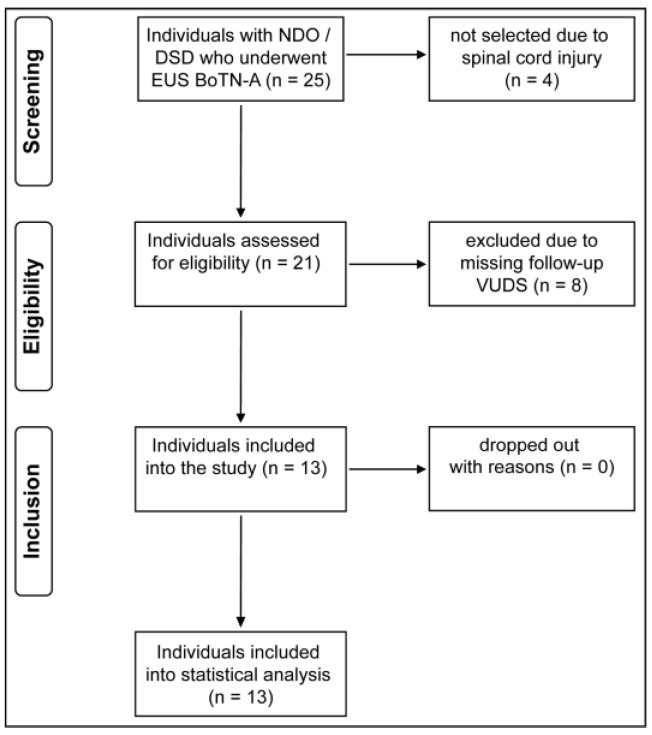
Study flow diagram. From a pool of 25 screened individuals, 13 fulfilled all inclusion criteria and were included into our analysis. BoNT-A = botulinum toxin-A, DSD = detrusor sphincter dyssynergia, EUS = external urethral sphincter, NDO = neurogenic detrusor overactivity, and VUDS = video-urodynamic studies.

Data were collected between 2015 and 2021 (Figure 1). We retrospectively obtained data from medical charts (i.e., categorical and continuous outcome variables, as well as posttreatment complications). (V)UDS were conducted using the urodynamic system (Uromic Quickstep, Medkonsult Medical Technology, MMT; Olomouc, Czech Republic) in combination with a fluoroscopy system (Artis zee multi-purpose, Siemens Healthineers International AG; Erlangen, Germany). The Uromic Quickstep system comprises pelvic floor muscles (PFM)/external anal sphincter (EAS) electromyography (EMG) to record PFM/EAS activity during (V)UDS. All urodynamic assessments were performed in accordance with the International Continence Society’s ‘Good Urodynamic Practices’ to evaluate lower urinary tract (LUT) function and quantify the current extent of neurogenic lower urinary tract dysfunction (NLUTD) [17].

### 2.5. Botulinum Toxin-A Injection

The BoNT-A was administered transcutaneously through a co-axial needle with simultaneous EMG control (Dantec^TM^ Clavis^TM^ device for EMG-guided injections with Bo-ject^®^ hypodermic needle electrode, Middleton, WI, USA) as a single transperineal injection into the EUS under digital rectal guidance. In accordance with Gallien et al. [9], the co-axial needle was inserted perineal in the midline about 1–2 cm above the anal margin.

### 2.6. Bias

We encountered two kinds of bias: (1) selection bias—all the patients were retrieved retrospectively during a pre-defined 6-year period, and (2) temporal bias—the time point of VUDS across all the patients was not standardized and, therefore, comprised a significant variance.

### 2.7. Statistical Methods

Statistical analyses were conducted using R (Version 4.0.5 for Mac Os). Non-parametric statistics were applied. Data are presented as raw values and percentages, medians with lower (Q1) and upper quartiles (Q3), as well as minimum and maximum, wherever indicated. Wilcoxon rank sum test was used for comparing continuous variables.

## 3. Results

In total, 13 patients (all male, median age 31 years) with a variety of underlying neurological disorders were included (see Table 1). Eight patients had a reduced vigilance, while the remaining five were fully alert (i.e., GCS = 15). In addition, nine (69%) patients had a percutaneous endoscopic gastrostomy (PEG) in place and five (38%) had a tracheostomy.

Seven patients received 100 units of BoNT-A, whereas the other six received 150 units. The median time from initial VUDS to BoNT-A injections was 28 days. The median time between BoNT-A injection and follow-up (V)UDS was 50 days [35; 70], 10–123.

There was a significant reduction in Pdetmax (*p* = 0.006, see Table 2) posttreatment. Although DOLPP was reduced in nine patients (69%) after treatment, there was no statistically significant difference compared to baseline. Both maximum cystometric capacity (190 vs. 220 mL) and leak volume (120 vs. 220 mL) were increased posttreatment, yet were not statistically significant.

While seven patients (54%) had indwelling catheters in place before the BoNT-A injection, all patients were able to empty their bladder sufficiently with reflex micturition (i.e., NDO incontinence) posttreatment. With respect to adverse events post-surgery, no complications, including local hematomas or infections, were reported.

Ten patients (77%) underwent a second BoNT-A injection, the median time between injection 1 and 2 was 184 days [112; 230, 97–260], while one patient was lost to follow-up. Of the two remaining patients, one patient underwent the insertion of a urethral stent (31 years old, UWS following TBI), while the other had a transurethral resection of the prostate combined with a bladder neck incision (32 years old, with a GCS = 15 following stroke)—both three months later.

## 4. Discussion

### 4.1. Main Findings

Our retrospective exploratory study revealed that intrasphincteric EMG-guided perineal BoNT-A injections in non-SCI patients with DSD are safe and reduced bladder outlet obstruction to a certain degree, in that all patients in this cohort were free of indwelling urinary catheters posttreatment.

### 4.2. Findings in the Context of the Existing Literature

NDO and DSD are both very prevalent in individuals with suprasacral SCI after the initial spinal shock phase (i.e., ~95% [18] and >70% [19]) and to a lesser degree in individuals with MS (i.e., ~43% and ~36%) [20].

With respect to the treatment of DSD, several studies have previously reported on the efficacy of intrasphincteric BoNT-A injections in patients with SCI [9,10,11,12,13,14,15] or MS [16]. To the best of our knowledge, this study highlights novel insights on the efficacy of intrasphincteric BoNT-A injections in patients without spinal cord lesions. Considering the lack of evidence in patients with non-SCI related DSD, we compared the efficacy of intrasphincteric BoNT-A injections in our cohort to those with SCI and MS.

PVR urine volume was the most frequent outcome measure in the aforementioned studies with SCI [9,10,11,12,13,14,15] and MS [16]. In line with our study, Gallien et al. [9] did not find a statistically significant change posttreatment. All remaining studies comprising patients with SCI reported a statistically significant (*p* < 0.05) lower PVR urine volume, either one [1,11,13,14,15] or three months [12] posttreatment. Although Gallien et al. [16] did not report a direct pre-/posttreatment comparison in patients with MS, a mean reduction (−34 mL) in PVR urine volume was noted posttreatment.

Pdetmax was another outcome measure in a few studies, i.e., in patients with SCI [9,11,13,14] and MS [16]. Three [9,13,14] of four studies comprising patients with SCI, as well as Gallien et al. [16], in patients with MS (−15 cmH_2_O) reported a reduction in Pdetmax posttreatment. However, in line with our findings, only Gallien et al. [9] highlighted a statistically significant (*p* < 0.05) lower Pdetmax after intrasphincteric BoNT-A injections.

Lastly, ‘detrusor leak point pressure’ [13,14] and ‘bladder pressure on voiding’ [10], as well as ‘maximum bladder capacity’ (i.e., MCC) [16] and ‘detrusor compliance at functional bladder capacity’ (i.e., compliance) [16], were less frequently reported outcome measures. While Petit et al. [10] had a statistically significant (*p* < 0.01) reduction in ‘bladder pressure on voiding’, Chen et al. [13,14] did not find a significant change in ‘detrusor leak point pressure’ posttreatment. In line with Gallien et al. [16] (i.e., mean +11 mL and +13 mL/cmH_2_O in patients with MS), we observed posttreatment increases (i.e., median +30 mL and +16 mL/cmH_2_O) in MCC and compliance, respectively. However, these changes did not yield statistical significance.

Indwelling urinary catheters are a risk factor for urinary tract infections [21]. Further, catheter-associated urinary tract infections (CAUTI) account for 40% of all hospital-acquired infections in the US and are therefore the most common type of nosocomial infection [22]. At baseline, seven patients were relying on indwelling catheters as their primary bladder-emptying method. By reducing functional outlet obstruction successfully, all seven patients were able to initiate reflex micturition and use urinary condoms to collect urine. Thus, no patient further relied on an indwelling catheter posttreatment.

Intrasphincteric BoNT-A injections can be performed either via a transperineal or transurethral approach. While Chen et al. [23] reported equivalent functional results with either approach, Schurch et al. [8] were able to demonstrate, with the aid of magnetic resonance imaging, that EMG-guided transperineal injections of Gadopentate-marked BoNT-A are precisely locatable in the EUS [8]. In our study, the transperineal approach was chosen because of its less invasive nature by avoiding an endoscopic procedure in these severely handicapped patients. Further, transurethral injections often require general or spinal anesthesia [14].

The therapeutic effect after one intrasphincteric BoNT-A injection lasts for about 2–3 months [12]. Thereafter, bladder outlet obstruction will reoccur. A therapeutic alternative to intrasphincteric BoNT-A injections long-term are urethral sphincter stents. Pannek et al. showed their safety and feasibility in patients with SCI after failed surgical sphincterotomy [24], but this method is associated with complications such as stent dislocation or stent stenosis [25,26]. However, evidence for treatment efficacy regarding urethral sphincter stents in patients such as in our cohort is lacking as well.

### 4.3. Limitations

Our study has various limitations. Considering the retrospective nature of our study [27], we did not perform follow-up (V)UDS systematically (i.e., following a pre-defined protocol and pre-specified time point) following intrasphincteric BoNT-A injections. Regarding follow-up (V)UDS, our institute has started to systematically perform these after intrasphincteric BoNT-A injections, i.e., between 1 and 2 months, in order to limit temporal bias. In addition, two different BoNT-A dosages were applied within our cohort. Further, there was a limited number of included patients with heterogeneity with respect to the underlying neurological disorders. One potential way of overcoming these limitations is to standardize this procedure. In addition, we cannot exclude a synergistic effect through polypharmacy, as we did not exclude patients on aiding meds (e.g., alpha blockers).

## 5. Conclusions

EMG-guided transperineal BoNT-A injections into the EUS appear to be a feasible and safe treatment option in patients with non-SCI DSD. However, given the paucity of data and the aforementioned limitations, well-designed prospective multi-institutional studies are necessary in order to allow a general, evidence-based recommendation regarding the relevance of intrasphincteric BoNT-A injections in patients with non-SCI DSD.

## Figures and Tables

**Table 1:** Patient demographics, injury characteristics, and treatment.

Demographics	Results
Age (years)	31 [24; 43], 19–53
Sex (male)	13 (100%)
**Injury characteristics**	
Type of brain injury	
*Traumatic*	*6 (46.2%)*
*Hypoxic*	*3 (23.1%)*
*Stroke*	*1 (7.7%)*
*Cerebral palsy*	*1 (7.7%)*
*Williams Beuren Syndrome*	*1 (7.7%)*
*Multiple sclerosis without spinal cord lesion*	*1 (7.7%)*
Vigilance	
*Normal (i.e., GCS = 15)*	*5 (39.0%)*
*UWS*	*4 (30.5%)*
*MCS*	*4 (30.5%)*
**Treatment**	
Botulinum toxin-A	
*100 units*	*7 (54%)*
*150 units*	*6 (46%)*

Results are shown as median, lower and upper quartiles, and range for age or number (%). Glasgow Coma Scale (GCS), unresponsive wakefulness syndrome (UWS) or minimally conscious state (MCS).

**Table 2:** Video-urodynamic (VUDS) parameters and bladder management—pre- vs. posttreatment.

**Urodynamic Parameters**	**1st VUDS (n = 13)**	**2nd (V)UDS (n = 13)**
NDO (yes/no)	13 (100%)	12 (92%)/1 (8%)
Compliance mL/cmH_2_O	34 [23; 54], 8–179	50 [33; 76], 15–110
DOLPP (cmH_2_O)	50 [40; 78], 34–126	50 [36; 57], 30–90
Leak volume (mL)	120 [70; 180], 0–360	220 [68; 374], 30–572
Pdetmax during storage (cmH_2_O) *	105 [85; 113], 44–143	54 [49; 70], 37–100
Pdetmax during storage > 40 cmH_2_O (yes or no)	13 (100%)/0	12 (92%)/1 (8%)
MCC (mL)	190 [120; 275], 60–360	220 [108; 336], 30–770
PVR urine (yes/no)	7 (54%)/6 (46%)	7 (54%)/6 (46%)
PVR urine (mL)	0 [0; 46], 0–160	0 [0; 150], 0–323
DSD (yes/no)	13 (100%)	10 (77%)/3 (23%)
**Bladder-emptying methods**	**Pre-treatment (n = 13)**	**Posttreatment (n = 13)**
Indwelling urinary catheters (yes/no)	7 (54%)	0
*Transurethral*	*4*	*0*
*Suprapubic*	*3*	*0*
Intermittent catheterization	0	0
NDO incontinence	6 (46%)	13 (100%)
Volitional voiding	0	0

DOLPP = Detrusor overactivity leak point pressure, DSD = detrusor sphincter dyssynergia, MCC = maximal cystometric capacity, NDO = neurogenic detrusor overactivity, Pdetmax = maximal detrusor pressure, PVR = post-void residual, * *p* value < 0.05. Results are shown as median, lower and upper quartiles, and range for compliance, DOLPP, Leak volume, Pdet max during storage, MCC, PVR urine.

## Data Availability

De-identified data are available upon request from the authors.
